# Age-, Sex-, and Ancestry-Specific Prevalence of Hearing Loss in UK Biobank and All of Us Research Program

**DOI:** 10.14336/AD.2025.0754

**Published:** 2026-07-09

**Authors:** Jun He, Sharon G. Curhan, Gary C. Curhan, Renato Polimanti

**Affiliations:** ^1^Department of Psychiatry, Yale University School of Medicine, New Haven, CT 06510, USA.; ^2^Veteran Affairs Connecticut Healthcare System, West Haven, CT 06516, USA.; ^3^Channing Division of Network Medicine, Department of Medicine, Brigham and Women's Hospital, Boston, MA 02115, USA.; ^4^Department of Medicine, Harvard Medical School, Boston, MA 02115, USA.; ^5^Department of Chronic Disease Epidemiology, Yale School of Public Health, New Haven, CT 06511, USA.; ^6^Wu Tsai Institute, Yale University, New Haven, CT 06511, USA.

**Keywords:** Hearing Loss, Aging, Population Prevalence, Sex Differences

## Abstract

Hearing loss (HL) is a leading cause of global disease burden, but limited information is available regarding differences among individuals of diverse ancestral backgrounds. Here, we assessed age-, sex-, and ancestry-specific prevalence of HL in 827 406 participants from UK Biobank (UKB, N = 448 193) and All of Us Research Program (AoU, N = 379 213). HL was defined based on electronic health records and self-reported information, and HL prevalence was calculated for each subgroup. Age trends and prevalence differences between sexes and ancestries were tested. Age-standardized prevalence was computed for UKB and AoU using the combined sample size of the two cohorts as the standard population. The overall HL prevalence was 28% in UKB (24% in females; 34% in males) and 16% in AoU (13% in females; 20% in males). Sex differences were statistically significant across all age groups in UKB, and among participants aged >55 years in AoU. Across ancestry groups, males had a higher prevalence of HL than females, except for those of African descent in the AoU sample (10% in females and 9.5% in males). The ancestry-specific prevalence was highest among those of European descent in both UKB (29%) and AoU (20%), and lowest among those of African descent (12%) in UKB and Central/South Asian descent (7.3%) in AoU. The HL polygenic risk score was associated with HL in both female and male samples. Overall, this study provides comprehensive evidence that sex differences in HL prevalence should be considered in the context of population diversity.

## INTRODUCTION

Hearing ability declines with age [1]. In 2019, hearing loss (HL) was the number one cause of years lived with disability (YLDs) among adults 70 years old and older [2]. HL can lead to social isolation and is associated with numerous health issues, consequently reducing the quality of life for older adults and imposing a significant economic burden on society [3]. Studies have investigated the prevalence of HL across different sex, race/ethnicity, and age groups [4]. However, these findings were primarily based on self-reported race/ethnicity, with few studies evaluating HL according to genetically informed ancestry across diverse populations. Additionally, most studies were performed in cohorts with a relatively limited sample size.

In this study, leveraging UK Biobank (UKB) [5] and All of Us Research Program (AoU) [6] with far larger sample sizes than previous reports, we assessed the age- and sex-specific prevalence of HL and examined how it varied across genetically informed ancestry groups. Employing both EHR and self-reported data, we present epidemiological estimates of the prevalence of HL among middle-aged and older adults in the UK and the US. We also explored the association between HL and its polygenic risk score (PRS) to assess the role of genetic predisposition in HL sex differences. The findings generated provide foundational evidence for future research to understand the risks of HL across population groups.

## METHODS

### Data sources

The data of the present study were extracted from two national biobanks. UKB is a large population-based cohort including phenotypic and genetic data from >500, 000 participants in the United Kingdom [5]. AoU is an ongoing nationwide prospective cohort study in the United States with more than 574 000 individuals recruited to date [6].

### Hearing loss assessment

In UKB, we assessed HL using three items: conductive and sensorineural HL (Field ID 131258) and other HL (Field ID 131260) from first occurrences data and self-reported information on hearing difficulty/problems (Field ID 2247). In AoU, we extracted information from its Curated Data Repository version 8 to determine HL, including HL (Concept ID 377889) and hearing problem (Concept ID 4101199) from Electronic Health Records, deaf or serious difficulty hearing (Concept ID 903573) from the Basics survey, and severe HL or partial deafness (Concept ID 1384396) from Personal and Family Health History survey. Details of these variables are provided in Supplementary Table 1. We stratified both cohorts based on sex, age, and genetically informed ancestry. Age was categorized as <45, 45-49, 50-54, 55-59, 60-64, and ≥65 years (y) old. For ≥65y group, additional stratification was performed after combining ancestry groups with small sample sizes. Leveraging genetically inferred classifications, UKB and AoU participants were clustered into six ancestry groups: Admixed American (AMR), African (AFR), Central/South Asian (CSA), East Asian (EAS), European (EUR), and Middle Eastern (MID). In UKB, ancestry assignments were based on principal components analysis (PCA) conducted on reference panels including the Human Genome Diversity Panel (HGDP) and the 1000 Genomes Project. UKB participants were projected into this PCA space using their genotypes and subsequently classified into six ancestry groups using the random forest algorithm (probability > 0.5) [7]. In AoU, a similar PCA strategy was employed based on the individual whole-genome sequencing data [8]. The two cohort approaches are broadly comparable in reference to populations, dimensionality reduction, and supervised classification.

### Statistical analysis

We calculated sex- and ancestry-stratified HL prevalence and 95% confidence intervals in the overall samples and in the age groups available from UKB and AoU cohorts. As previously proposed [9, 10], we considered the combination of UKB and AoU participants as the standard population to compute the age-standardized prevalence for each subgroup. Statistical tests were performed to assess prevalence differences between participant subgroups. Specifically, Mantel-Haenszel Chi-squared test was applied to compare overall prevalence across sexes, ancestries, and cohorts after adjusting for the effect of age groups. With regard to the age-specific prevalence, Chi-square test was performed when the expected frequency in each cell was at least five. Otherwise, Fisher’s exact test was used. For sex differences, we conducted the age-specific test only for ancestries that showed statistical significance in the age-combined analysis. Additionally, Chi-square test for trend in proportions was used to assess whether the prevalence increased with age. For All above tests, statistical significance was defined applying Bonferroni multiple testing correction, and all data were analyzed and visualized using R (version 4.3.2). Using the PRS-CS approach [11] and AoU genetic data, we further investigated the sex-specific association of AoU HL with the PRS of HL derived from previously meta-analyzed genome-wide association statistics, which were mainly based on UKB participants [12]. The goals of this analysis were to evaluate the external transferability of genetic predisposition to HL across cohorts and to compare its associations between sexes. The PRS analysis was limited to EUR ancestry, and logistic regression was applied to assess the association including age and the top 10 within-ancestry principal components as covariates. In an additional sensitivity analysis, we calculated the sample overlap between different UKB data sources and estimated HL prevalence only based on the first occurrence data in UKB.

## RESULTS

Our analysis included a total of 827 406 participants (58% females; mean age 56y) from UKB (54% females; mean age 57y) and AoU (62% females; mean age 55y; Supplementary Table 2). The age distributions differed between the two cohorts, with 30% of participants in AoU younger than 45y, compared to only 10% in UKB. The combined sample included 22% participants of non-European descent but differed substantially between the two cohorts (4.8% in UKB; 41% in AoU).

**Figure 1.**
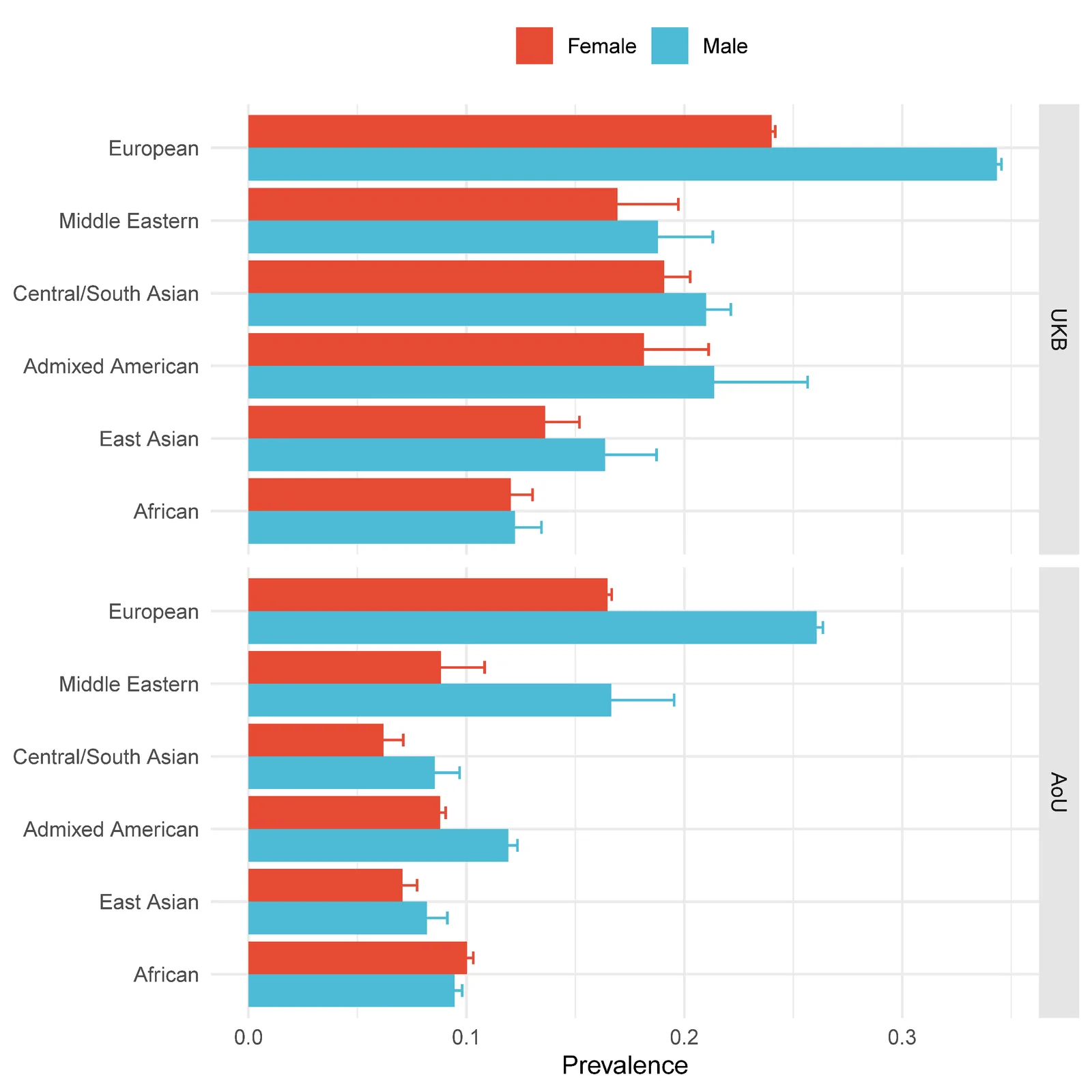
**Sex- and ancestry-specific prevalence of hearing loss in UK Biobank (UKB) and All of Us Research Program (AoU)**.

In UKB, HL prevalence in the overall sample was 28%, with 24% and 34% in females and males, respectively. Considering the overall UKB sample, HL prevalence was statistically different across ancestries (*P* = 1.3×10^-207^; Supplementary Table 3 and 4), with the highest estimate in EUR (29%) and the lowest in AFR (12%). In EUR, females had a lower prevalence than males (Female 24% vs. Male 34%, *P* < 1.0×10^-300^; Fig. 1, Supplementary Table 3 and 5). Likely because of the limited diversity of UKB cohort, sex differences were not statistically significant in the other ancestry groups (Supplementary Table 5). In AoU, HL prevalence was 16% in the overall sample, 13% in females, and 20% in males. Differences across ancestries in AoU were also significant (Supplementary Table 4), ranging from 20% in EUR to 7.3% in CSA. AoU-AFR (9.8%) was also much lower than AoU-EUR. In the more diverse AoU sample, we observed sex differences in multiple population groups (Fig. 1, Supplementary Table 5). In AMR, MID, and EUR, male HL prevalence was higher than that of females (e.g., MID: 17% vs. 8.8%, *P* = 0.001). Conversely, in AoU-AFR, female HL prevalence was slightly higher than that of males (10.0% vs. 9.5%, *P* = 1.8×10^-7^). Compared to UKB, AoU participants showed a statistically lower HL prevalence in both sex-combined and sex-stratified analyses (Supplementary Table 3 and 6). In PRS analysis, the HL PRS was strongly associated with HL both in females (OR = 1.2, *P* = 3.8×10^-76^) and males (OR = 1.2, *P* = 1.0×10^-62^) in AoU.

**Figure 2.**
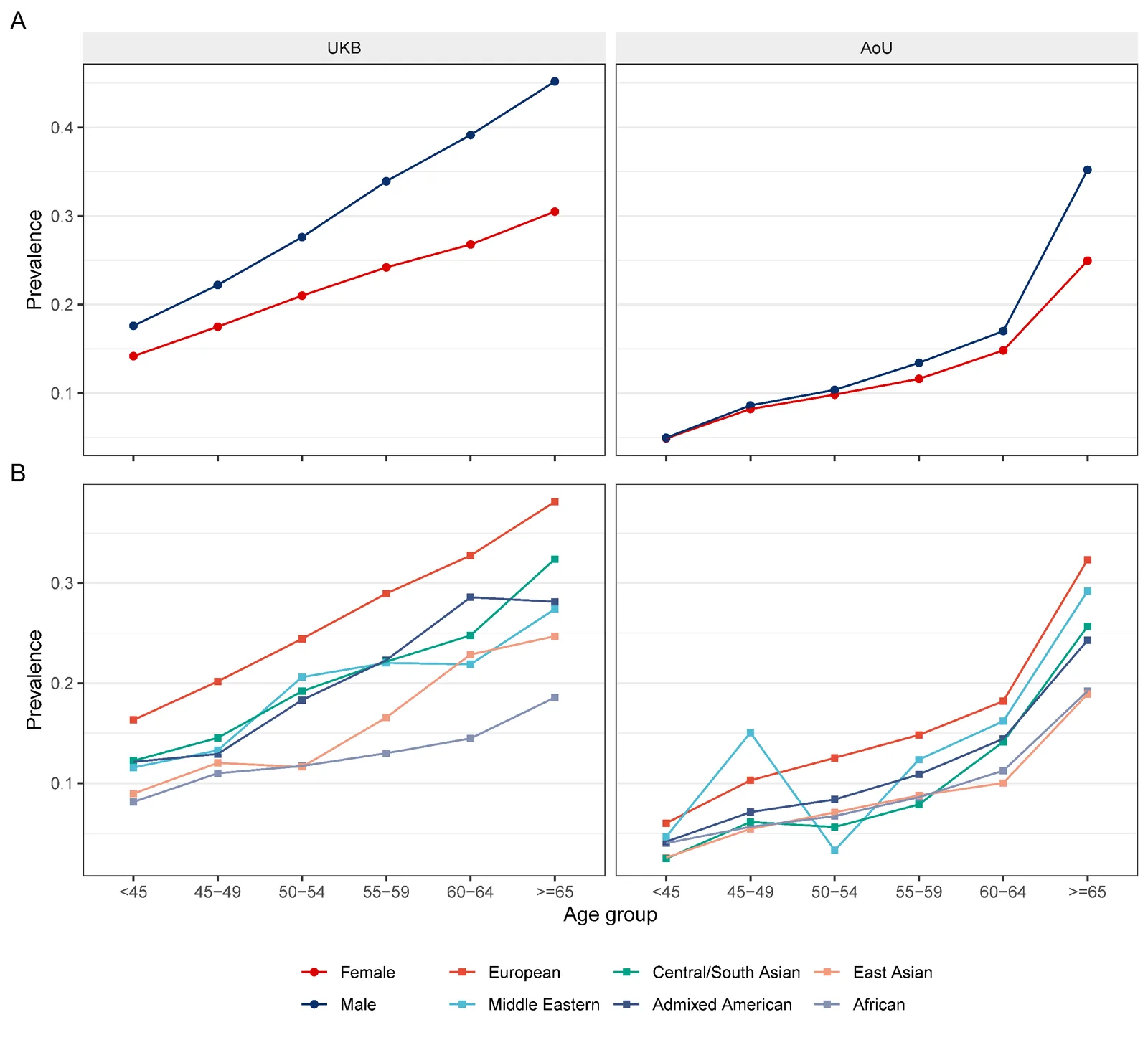
**Age-trends in the prevalence of hearing loss by sex (A) and ancestry (B) in UK Biobank (UKB) and All of Us Research Program (AoU)**.

The patterns observed in the crude-prevalence analyses were in line with the age-standardized estimates (Supplementary Table 3). In the analysis stratified by age groups, HL prevalence increased in older participants in both UKB (16% to 38%, *P* for trend < 1.0×10^-300^) and AoU (4.9% to 30%, *P* for trend < 1.0×10^-300^; Supplementary Table 7 and 8). In UKB, HL prevalence was higher in males than in females across all age groups (*P* ≤ 3.5×10^-23^), while AoU sex differences were observed only in older groups (≥55y; *P* ≤ 5.0×10^-7^) (Fig. 2, Supplementary Table 5 and 7). In UKB, EUR had the highest prevalence across six age groups in males (Table 1, Supplementary Fig. 1, Supplementary Table 4 and 7), with nearly half (47%) of those ≥70y experiencing HL (Supplementary Table 9). Conversely, the ancestry differences were less marked in females (Table 1, Supplementary Fig. 1, Supplementary Table 4 and 7). In AoU, excluding MID due to its small sample size, the highest HL prevalence for all age groups was observed in EUR (Fig. 2, Supplementary Table 4 and 7), with 58% for EUR males ≥85y (Supplementary Table 9).

In AoU-EUR, males had higher HL prevalence than females only in adults ≥50y (*P* ≤ 2.4×10^-4^). Comparing the estimates observed between the two cohorts, the prevalence of HL as assessed in UKB was higher than the prevalence of HL as assessed in AoU with respect to all age groups in overall sample, females, and males (Supplementary Table 6 and 7). In the sensitivity analysis to explore possible differences across data sources, 74% of HL cases identified from first occurrences and 85% cases from hospital inpatient records in UKB also overlapped with self-reported cases. When restricting UKB definitions to first occurrence data only, the observed sex- and ancestry-differences remained largely unchanged (Supplementary Table 10).

**Table 1:** Age-, sex-, and ancestry-specific prevalence (%) of hearing loss in UK Biobank (UKB) and All of Us Research Program (AoU).

Age, years	Hearing Loss, ^a^ %											
**UKB**	**Female**						**Male**					

	**EUR** **(n = 230868)**	**MID** **(n = 691)**	**CSA** **(n = 4193)**	**AMR** **(n = 645)**	**EAS** **(n = 1836)**	**AFR** **(n = 4015)**	**EUR** **(n = 196012)**	**MID** **(n = 931)**	**CSA** **(n = 4915)**	**AMR** **(n = 351)**	**EAS** **(n = 947)**	**AFR** **(n = 2789)**

<45	14.5	11.8	13.0	11.5	9.7	7.5	18.4	11.4	11.6	13.1	7.8	8.9
45-49	17.9	13.4	15.0	12.9	10.8	11.7	23.0	13.2	14.2	13.0	14.3	9.9
50-54	21.3	21.8	21.8	15.6	12.1	12.1	28.4	19.6	16.6	25.0	10.6	11.2
55-59	24.5	18.6	19.2	21.3	16.3	12.3	34.5	24.7	24.8	25.7	17.4	14.0
60-64	27.0	22.1	22.0	24.0	18.1	14.2	39.5	21.7	27.3	35.2	31.5	14.9
≥65	30.7	16.7	27.0	32.1	21.3	17.8	45.6	34.9	35.7	22.5	30.0	19.5

**AoU**	**EUR** **(n = 134715)**	**MID** **(n = 770)**	**CSA** **(n = 2711)**	**AMR** **(n = 46672)**	**EAS** **(n = 5669)**	**AFR** **(n = 43657)**	**EUR** **(n = 88086)**	**MID** **(n = 637)**	**CSA** **(n = 2316)**	**AMR** **(n = 23117)**	**EAS** **(n = 3262)**	**AFR** **(n = 27601)**

<45	6.0	3.8	2.4	3.9	2.8	4.1	6.0	5.9	2.5	4.7	2.2	3.8
45-49	9.9	12.3	6.8	6.9	5.5	6.1	11.0	18.8	5.3	7.6	5.2	4.8
50-54	11.8	3.6	6.3	7.8	7.7	7.6	13.9	2.9	4.9	9.4	6.1	5.3
55-59	13.3	7.5	8.6	10.4	9.8	9.1	17.6	19.4	7.1	11.7	7.1	7.9
60-64	16.4	16.7	14.7	13.9	9.0	11.9	21.0	15.7	13.5	15.4	11.8	10.4
≥65	26.7	21.0	20.5	21.5	17.4	19.8	38.8	35.8	29.7	28.7	21.0	18.4

EUR: European; MID: Middle Eastern; CSA: Central/South Asian; AMR: Admixed American; EAS: East Asian; AFR: African.

a Hearing loss was defined based on a combination of Electronic Health Records and self-reported data. Detailed descriptions of each cohort are provided in the METHODS section of the main text and in Supplementary Table 1.

## DISCUSSION

Investigating more than 800 000 participants from two countries, we observed age, sex- and ancestry-specific patterns of HL, which have substantial implications for designing interventions to reduce YLDs in the population, particularly among older individuals [2]. Building on previous evidence [13, 14], our analyses highlight that the higher HL prevalence in males compared to females differs across age groups, ancestries, and cohorts. Despite the sex differences in HL prevalence, the association of HL and its PRS did not differ between females and males. With respect to HL sex differences, increased occupational noise exposure in males has been previously indicated as a possible factor [13] in addition to biological mechanisms (e.g., estrogen levels and genetic predisposition) that may be protective in females [15, 16]. Similarly, differences in genetic variants and reporting rates may contribute to the higher HL prevalence in EUR compared to AFR [13, 17, 18]. With respect to these previous hypotheses, our findings provide new evidence. Firstly, we observed that, unlike all other ancestry groups tested in UKB and AoU, HL prevalence in AoU-AFR sample was statistically higher in females compared to males. This differs from previous investigations that used pure tone audiometry to define HL and further research is needed to identify the underlying reasons for these differences [19]. In addition, we observed that EUR has higher HL prevalence compared to multiple other ancestry groups and that the low HL prevalence in AFR may be influenced by cohort characteristics, such as demographic distributions, sample size, and HL assessment methods. These appear to also influence differences between sexes. Indeed, males had a consistently higher HL prevalence than females in UKB compared to AoU (e.g., AoU sex differences were statistically significant only among adults over 55y). Previous studies have shown that beyond environmental noise exposures, other factors clearly contribute to patterns of prevalence of HL (e.g., comorbid conditions, healthcare access, and sociocultural dynamics) [19, 20]. Also, different recruitment strategies, response rates, questionnaire designs, and data collection processes likely account for the observed differences in magnitude of HL prevalence between cohorts [5, 6]. For example, a large proportion of HL cases in AoU were identified through EHR data, which may partially explain the lower prevalence estimates, whereas most cases in UKB were derived from self-reported information. However, the high overlap of UKB HL cases between different data sources indicated certain consistency across data collection processes. Nevertheless, qualitative comparisons between cohorts should be interpreted with caution.

This study has several limitations. First, neither UKB nor AoU was specifically designed to estimate HL prevalence and therefore did not employ multistage random sampling to ensure representativeness. Nonetheless, the overall sample size is quite large and includes a wide range of age and ancestral groups. For certain ancestry groups (e.g., MID), the limited sample size may affect the precision of prevalence estimates. Although we conducted statistical tests for all subgroup comparisons, findings such as HL trends from underpowered groups should be interpreted cautiously. Second, both cohorts have a cross-sectional design, which precludes causal inferences regarding the age-related progression of HL. Third, potential biases may exist in the recruitment and survey processes, such as unequal response rates and reporting accuracy across subgroups [18]. Fourth, although most types of HL are sensorineural, we cannot distinguish them from congenital and conductive HL with self-reported information. Lastly, differences in HL ascertainment methods between the two cohorts and the use of internal standardization for prevalence estimates may limit the comparability of findings both between the cohorts and with external studies. In addition, neither cohort included pure tone audiometry, which may lead to misclassification of HL and underestimation of both the HL prevalence and the genetic effect sizes in the PRS analysis.

This large cross-sectional study found diverse HL patterns when examining the intersection of age, sex, and ancestry, highlighting the need to uncover the biological and environmental causes of these differences.

## Supplementary Materials

The Supplementary data can be found online at: www.aginganddisease.org/EN/10.14336/AD.2025.0754.



## Data Availability

The UK Biobank individual data could be obtained by applying on the website (http://www.ukbiobank.ac.uk/). The data of the All of Us Research Program used in this study were accessed from the All of Us Curated Data Repository version 8 (https://www.researchallofus.org).
