# Supplementary material for: Age-, Sex-, and Ancestry-Specific Prevalence of Hearing Loss in UK Biobank and All of Us Research Program
Source: Aging Dis. 2025 Jul 9;17(4):2253–9. doi: 10.14336/AD.2025.0754 (PMC13256383; doi:10.14336/AD.2025.0754)
Supplement: Supplementary file 1 [file AD-17-4-2253-s.pdf]

SUPPLEMENTARY DATA

Age-, Sex-, and Ancestry-Specific Prevalence of Hearing Loss in UK Biobank and All of Us Research Program

Jun He, Sharon G. Curhan, Gary C. Curhan, Renato Polimanti

© 2025. He J et al. Published online at http://www.aginganddisease.org/EN/10.14336/AD.2025.0754

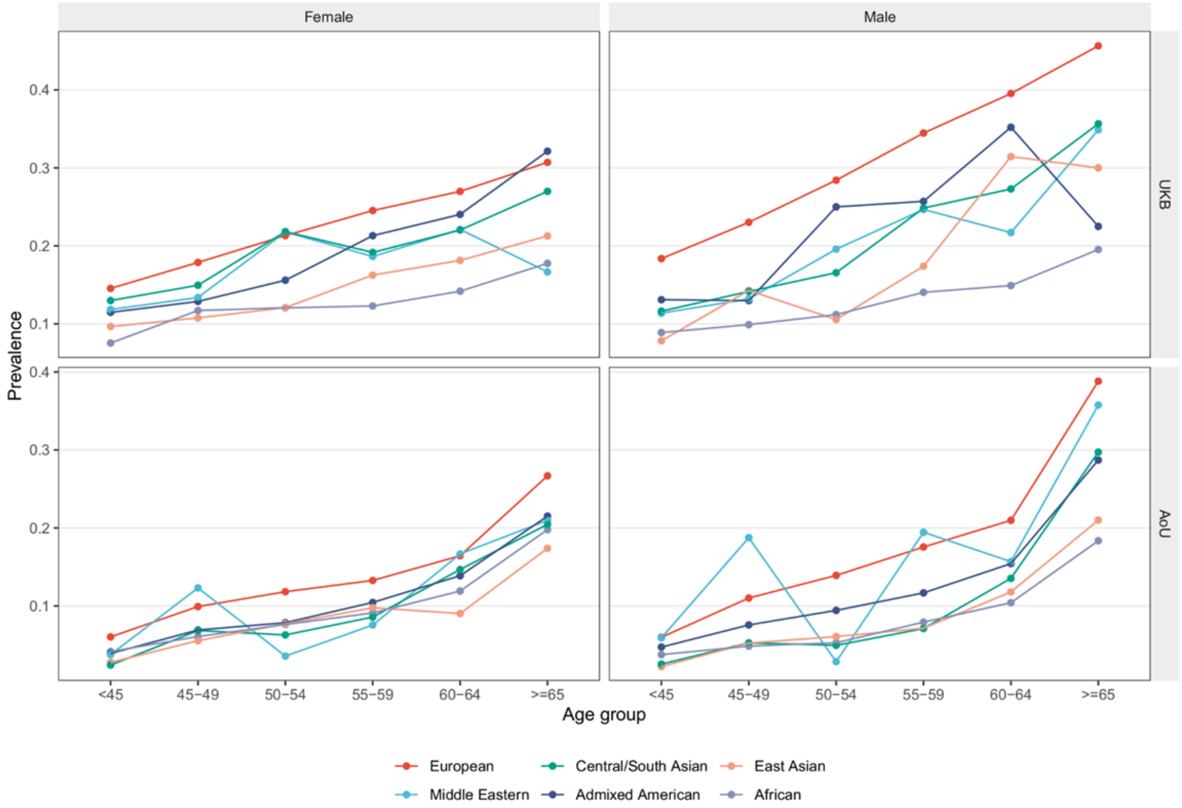

Supplementary Figure 1: Sex-, ancestry-, and age-specific hearing loss prevalence in UK Biobank (UKB) and All of Us Research Program (AoU).

Supplementary Table 1. Variables to assess hearing loss in UK Biobank (UKB) and All of Us Research Program (AoU).

Cohort	Category	Field ID/Concept ID	Variable name	Variable description
UKB	First occurrences	131258	Conductive and sensorineural hearing loss	Any code mapped to 3-character ICD10 H90, derived by combining primary care, hospital admission, death register, and self-reported data, was used to identify cases.
	First occurrences	131260	Other hearing loss	Any code mapped to 3-character ICD10 H91, derived by combining primary care, hospital admission, death register, and self-reported data, was used to identify cases.
	Self reported	2247	Hearing difficulty/problems	Individuals who selected "Yes" or "I am completely deaf" in response to the question "Do you have any difficulty with your hearing?" were identified as cases.
AoU	Electronic Health Records	377889	Hearing loss	Individuals with any of the following diagnoses in the Electronic Health Records were considered as cases: hard of hearing, difficulty hearing, hearing impairment, hearing impaired, impaired hearing, hearing loss (disorder), or Hypoacusis.

© 2025. He J et al. Published online at http://www.aginganddisease.org/EN/10.14336/AD.2025.0754

	Electronic Health Records	4101199	Hearing problem	Individuals with diagnosis of hearing problem in the Electronic Health Records were considered as cases.
	Basics survey	903573	Deaf or serious difficulty hearing	Individuals who selected "Yes" for the question "Are you deaf, or do you have serious difficulty hearing?" were identified as cases.
	Personal and Family Health History survey	1384396	Severe HL or partial deafness	This item applied a two-level self-reported question. First, individuals were asked a multiple-choice question: "Have you or anyone in your family ever been diagnosed with the following hearing and eye conditions? Think only of the people you are related to by blood. Select all that apply." If "Severe hearing loss or partial deafness in one or both ears" was selected from the optional conditions, a following question was asked: "Including yourself, who in your family has had severe hearing loss or partial deafness in one or both ears? Select all that apply." Individuals who selected "Self" were classified as cases.

Supplementary Table 2. Demographic characteristics of participants in UK Biobank and All of Us Research Program.

Characteristic, N (%)	UK Biobank			All of Us Research Program		
	Sex-combined	Female	Male	Sex-combined	Female	Male
**Total**	448 193 (100)	242 248 (54.0)	205 945 (46.0)	379 213 (100)	234 194 (61.8)	145 019 (38.2)
**Ancestry**						
Admixed American	996 (0.2)	645 (0.3)	351 (0.2)	69 789 (18.4)	46 672 (19.9)	23 117 (15.9)
African	6804 (1.5)	4015 (1.7)	2789 (1.4)	71 258 (18.8)	43 657 (18.6)	27 601 (19.0)
Central/South Asian	9108 (2.0)	4193 (1.7)	4915 (2.4)	5027 (1.3)	2711 (1.2)	2316 (1.6)
East Asian	2783 (0.6)	1836 (0.8)	947 (0.5)	8931 (2.4)	5669 (2.4)	3262 (2.2)
European	426 880 (95.2)	230 868 (95.3)	196 012 (95.2)	222 801 (58.8)	134 715 (57.5)	88 086 (60.7)
Middle Eastern	1622 (0.4)	691 (0.3)	931 (0.5)	1407 (0.4)	770 (0.3)	637 (0.4)
**Age**						
<45 years	44 970 (10.0)	24 003 (9.9)	20 967 (10.2)	116 196 (30.6)	78 667 (33.6)	37 529 (25.9)
45-49 years	57 849 (12.9)	32 036 (13.2)	25 813 (12.5)	26 206 (6.9)	17 546 (7.5)	8660 (6.0)
50-54 years	67 285 (15.0)	37 919 (15.7)	29 366 (14.3)	30 338 (8.0)	19 728 (8.4)	10 610 (7.3)
55-59 years	80 858 (18.0)	44 920 (18.5)	35 938 (17.5)	35 536 (9.4)	22 142 (9.5)	13 394 (9.2)
60-64 years	109 979 (24.5)	59 489 (24.6)	50 490 (24.5)	40 692 (10.7)	24 476 (10.5)	16 216 (11.2)
≥65 years	87 252 (19.5)	43 881 (18.1)	43 371 (21.1)	130 245 (34.3)	71 635 (30.6)	58 610 (40.4)

© 2025. He J et al. Published online at http://www.aginganddisease.org/EN/10.14336/AD.2025.0754

Supplementary Table 3. Prevalence of hearing loss in UK Biobank (UKB) and All of Us Research Program (AoU).

Cohort	Sex	Ancestry	Total	Hearing loss cases	Controls	Crude prevalence rate	SE	Lower 95%CI	Upper 95%CI	Age-standardized prevalence rate
UKB	Sex-combined	Ancestry-combined	448193	126259	321934	0.282	0.001	0.280	0.283	0.259
		Admixed American	996	192	804	0.193	0.012	0.168	0.217	0.218
		African	6804	824	5980	0.121	0.004	0.113	0.129	0.134
		Central/South Asian	9108	1832	7276	0.201	0.004	0.193	0.209	0.219
		East Asian	2783	405	2378	0.146	0.007	0.132	0.159	0.177
		European	426880	122714	304166	0.287	0.001	0.286	0.289	0.279
		Middle Eastern	1622	292	1330	0.180	0.010	0.161	0.199	0.197
	Female	Ancestry-combined	242248	57169	185079	0.236	0.001	0.234	0.238	0.223
		Admixed American	645	117	528	0.181	0.015	0.152	0.211	0.212
		African	4015	483	3532	0.120	0.005	0.110	0.130	0.131
		Central/South Asian	4193	800	3393	0.191	0.006	0.179	0.203	0.204
		East Asian	1836	250	1586	0.136	0.008	0.120	0.152	0.156
		European	230868	55402	175466	0.240	0.001	0.238	0.242	0.236
		Middle Eastern	691	117	574	0.169	0.014	0.141	0.197	0.173
	Male	Ancestry-combined	205945	69090	136855	0.335	0.001	0.333	0.338	0.305
		Admixed American	351	75	276	0.214	0.022	0.171	0.257	0.228
		African	2789	341	2448	0.122	0.006	0.110	0.134	0.139
		Central/South Asian	4915	1032	3883	0.210	0.006	0.199	0.221	0.235
		East Asian	947	155	792	0.164	0.012	0.140	0.187	0.203
		European	196012	67312	128700	0.343	0.001	0.341	0.346	0.333
		Middle Eastern	931	175	756	0.188	0.013	0.163	0.213	0.225
AoU	Sex-combined	Ancestry-combined	379213	60207	319006	0.159	0.001	0.158	0.160	0.166
		Admixed American	69789	6861	62928	0.098	0.001	0.096	0.101	0.133
		African	71258	6992	64266	0.098	0.001	0.096	0.100	0.104
		Central/South Asian	5027	366	4661	0.073	0.004	0.066	0.080	0.120
		East Asian	8931	668	8263	0.075	0.003	0.069	0.080	0.100
		European	222801	45146	177655	0.203	0.001	0.201	0.204	0.177
		Middle Eastern	1407	174	1233	0.124	0.009	0.106	0.141	0.149
	Female	Ancestry-combined	234194	31305	202889	0.134	0.001	0.132	0.135	0.146
		Admixed American	46672	4103	42569	0.088	0.001	0.085	0.090	0.120
		African	43657	4380	39277	0.100	0.001	0.098	0.103	0.110
		Central/South Asian	2711	168	2543	0.062	0.005	0.053	0.071	0.112
		East Asian	5669	401	5268	0.071	0.003	0.064	0.077	0.096
		European	134715	22185	112530	0.165	0.001	0.163	0.167	0.154
		Middle Eastern	770	68	702	0.088	0.010	0.068	0.108	0.120
	Male	Ancestry-combined	145019	28902	116117	0.199	0.001	0.197	0.201	0.189
		Admixed American	23117	2758	20359	0.119	0.002	0.115	0.123	0.148

© 2025. He J et al. Published online at http://www.aginganddisease.org/EN/10.14336/AD.2025.0754

		African	27601	2612	24989	0.095	0.002	0.091	0.098	0.097
		Central/South Asian	2316	198	2118	0.085	0.006	0.074	0.097	0.129
		East Asian	3262	267	2995	0.082	0.005	0.072	0.091	0.104
		European	88086	22961	65125	0.261	0.001	0.258	0.264	0.204
		Middle Eastern	637	106	531	0.166	0.015	0.137	0.195	0.184

Supplementary Table 4. Statistical test for ancestry differences in the prevalence of hearing loss. UKB: UK Biobank; AoU: All of Us Research Program.

Categorical variable tested	Cohort	Sex	Age group	Statistic	P value
Ancestry	UKB	Sex-combined	Adjusted	970.73	**1.3E-207**
			<45 years	109.88	**4.3E-22**
			45-49 years	150.33	**1.1E-30**
			50-54 years	194.74	**3.8E-40**
			55-59 years	189.02	**6.3E-39**
			60-64 years	180.45	**4.3E-37**
			≥65 years	168.28	**1.7E-34**
		Female	Adjusted	300.76	**6.9E-63**
			<45 years	37.47	**4.8E-07**
			45-49 years	44.44	**1.9E-08**
			50-54 years	64.22	**1.6E-12**
			55-59 years	70.00	**1.0E-13**
			60-64 years	56.46	**6.5E-11**
			≥65 years	47.47	**4.5E-09**
		Male	Adjusted	741.83	**4.4E-158**
			<45 years	82.20	**2.9E-16**
			45-49 years	120.29	**2.7E-24**
			50-54 years	162.98	**2.3E-33**
			55-59 years	122.10	**1.1E-24**
			60-64 years	137.47	**6.2E-28**
			≥65 years	143.72	**2.9E-29**
	AoU	Sex-combined	Adjusted	2685.30	**<1E-300**
			<45 years	307.63	**2.3E-64**
			45-49 years	146.31	**8.1E-30**
			50-54 years	233.26	**2.1E-48**
			55-59 years	251.57	**2.5E-52**
			60-64 years	286.61	**7.5E-60**
			≥65 years	1532.76	**<1E-300**
		Female	Adjusted	810.53	**6.1E-173**
			<45 years	218.37	**3.3E-45**
			45-49 years	71.86	**4.2E-14**
			50-54 years	97.18	**2.1E-19**
			55-59 years	73.49	**1.9E-14**
			60-64 years	81.45	**4.2E-16**
			≥65 years	316.01	**3.6E-66**
		Male	Adjusted	2061.65	**<1E-300**
			<45 years	102.70	**1.4E-20**
			45-49 years		**5.0E-04**
			50-54 years		**5.0E-04**
			55-59 years		**5.0E-04**
			60-64 years	250.53	**4.2E-52**
			≥65 years	1309.27	**6.3E-281**

© 2025. He J et al. Published online at http://www.aginganddisease.org/EN/10.14336/AD.2025.0754

Supplementary Table 5. Statistical test for sex differences in the prevalence of hearing loss. UKB: UK Biobank; AoU: All of Us Research Program.

Categorical variable tested	Cohort	Ancestry	Age group	Statistic	P value
Sex	UKB	Ancestry-combined	Adjusted	5189.32	**<1E-300**
			<45 years	98.37	**3.5E-23**
			45-49 years	201.31	**1.1E-45**
			50-54 years	396.83	**2.7E-88**
			55-59 years	924.66	**4.3E-203**
			60-64 years	1901.93	**<1E-300**
			≥65 years	2000.92	**<1E-300**
		Admixed American	Adjusted	1.44	0.230
		African	Adjusted	0.03	0.854
		Central/South Asian	Adjusted	3.19	0.074
		East Asian	Adjusted	3.75	0.053
		European	Adjusted	5279.36	**<1E-300**
		Middle Eastern	Adjusted	1.02	0.312
		European	<45 years	108.02	**2.7E-25**
			45-49 years	216.78	**4.6E-49**
			50-54 years	425.29	**1.7E-94**
			55-59 years	916.79	**2.2E-201**
			60-64 years	1899.52	**<1E-300**
			≥65 years	2003.38	**<1E-300**
	AoU	Ancestry-combined	Adjusted	1258.64	**1.1E-275**
			<45 years	0.24	0.624
			45-49 years	1.21	0.272
			50-54 years	2.09	0.148
			55-59 years	25.26	**5.0E-07**
			60-64 years	34.90	**3.5E-09**
			≥65 years	1627.49	**<1E-300**
		Admixed American	Adjusted	83.08	**7.9E-20**
		African	Adjusted	27.28	**1.8E-07**
		Central/South Asian	Adjusted	1.75	0.186
		East Asian	Adjusted	0.33	0.566
		European	Adjusted	1518.34	**<1E-300**
		Middle Eastern	Adjusted	10.30	**0.001**
		Admixed American	<45 years	11.22	**8.1E-04**
			45-49 years	0.77	0.380
			50-54 years	4.54	0.033
			55-59 years	2.22	0.136
			60-64 years	2.72	0.099
			≥65 years	78.76	**7.0E-19**
		African	<45 years	1.81	0.178
			45-49 years	3.44	0.064
			50-54 years	14.39	**1.5E-04**
			55-59 years	3.83	0.050
			60-64 years	5.54	0.019
			≥65 years	5.50	0.019
		European	<45 years	0.04	0.847
			45-49 years	3.60	0.058
			50-54 years	13.48	**2.4E-04**
			55-59 years	62.33	**2.9E-15**
			60-64 years	75.76	**3.2E-18**
			≥65 years	1638.29	**<1E-300**
		Middle Eastern	<45 years	1.28	0.258
			45-49 years	0.46	0.496

© 2025. He J et al. Published online at http://www.aginganddisease.org/EN/10.14336/AD.2025.0754

			50-54 years		1.000
			55-59 years		0.112
			60-64 years	0.00	1.000
			≥65 years	7.70	0.006

Supplementary Table 6: Statistical test for cohort differences in the prevalence of hearing loss between UK Biobank and All of Us Research Program.

Categorical variable tested	Ancestry	Sex	Age group	Statistic	P value
Cohort	Ancestry-combined	Sex-combined	Adjusted	15724.24	**<1E-300**
			<45 years	5232.10	**<1E-300**
			45-49 years	1693.07	**<1E-300**
			50-54 years	2557.41	**<1E-300**
			55-59 years	3609.16	**<1E-300**
			60-64 years	4146.52	**<1E-300**
			≥65 years	1601.55	**<1E-300**
		Female	Adjusted	6107.98	**<1E-300**
			<45 years	2417.60	**<1E-300**
			45-49 years	802.15	**1.8E-176**
			50-54 years	1139.36	**9.2E-250**
			55-59 years	1466.82	**<1E-300**
			60-64 years	1387.59	**1.0E-303**
			≥65 years	422.72	**6.3E-94**
		Male	Adjusted	8718.85	**<1E-300**
			<45 years	2502.32	**<1E-300**
			45-49 years	784.79	**1.1E-172**
			50-54 years	1308.46	**1.6E-286**
			55-59 years	2017.55	**<1E-300**
			60-64 years	2688.86	**<1E-300**
			≥65 years	1037.09	**1.6E-227**

© 2025. He J et al. Published online at http://www.aginganddisease.org/EN/10.14336/AD.2025.0754

Supplementary Table 7. Age-specific prevalence of hearing loss in UK Biobank (UKB) and All of Us Research Program (AoU).

Cohort	Sex	Ancestry	Age group (years)	Hearing loss cases	Controls	Prevalence rate	SE
UKB	Sex-combined	Ancestry-combined	<45	7093	37877	0.158	0.002
			45-49	11340	46509	0.196	0.002
			50-54	16077	51208	0.239	0.002
			55-59	23065	57793	0.285	0.002
			60-64	35701	74278	0.325	0.001
			≥65	32983	54269	0.378	0.002
		Admixed American	<45	25	181	0.121	0.023
			45-49	27	182	0.129	0.023
			50-54	28	125	0.183	0.031
			55-59	35	122	0.223	0.033
			60-64	50	125	0.286	0.034
			≥65	27	69	0.281	0.046
		African	<45	107	1209	0.081	0.008
			45-49	176	1426	0.110	0.008
			50-54	169	1273	0.117	0.008
			55-59	118	790	0.130	0.011
			60-64	110	650	0.145	0.013
			≥65	144	632	0.186	0.014
		Central/South Asian	<45	210	1506	0.122	0.008
			45-49	239	1406	0.145	0.009
			50-54	312	1314	0.192	0.010
			55-59	345	1213	0.221	0.011
			60-64	338	1027	0.248	0.012
			≥65	388	810	0.324	0.014
		East Asian	<45	51	518	0.090	0.012
			45-49	73	534	0.120	0.013
			50-54	61	464	0.116	0.014
			55-59	83	418	0.166	0.017
			60-64	80	270	0.229	0.022
			≥65	57	174	0.247	0.028
		European	<45	6658	34142	0.163	0.002
			45-49	10778	42654	0.202	0.002
			50-54	15452	47820	0.244	0.002
			55-59	22425	55041	0.289	0.002
			60-64	35074	72031	0.327	0.001
			≥65	32327	52478	0.381	0.002
		Middle Eastern	<45	42	321	0.116	0.017
			45-49	47	307	0.133	0.018
			50-54	55	212	0.206	0.025
			55-59	59	209	0.220	0.025
			60-64	49	175	0.219	0.028
			≥65	40	106	0.274	0.037
	Female		<45	3403	20600	0.142	0.002

© 2025. He J et al. Published online at http://www.aginganddisease.org/EN/10.14336/AD.2025.0754

		Ancestry-combined	45-49	5606	26430	0.175	0.002
			50-54	7967	29952	0.210	0.002
			55-59	10873	34047	0.242	0.002
			60-64	15936	43553	0.268	0.002
			≥65	13384	30497	0.305	0.002
		Admixed American	<45	<20	<200	0.115	0.029
			45-49	<20	<200	0.129	0.029
			50-54	<20	<100	0.156	0.035
			55-59	26	96	0.213	0.037
			60-64	25	79	0.240	0.042
			≥65	<20	<100	0.321	0.062
		African	<45	56	686	0.075	0.010
			45-49	112	844	0.117	0.010
			50-54	106	773	0.121	0.011
			55-59	67	478	0.123	0.014
			60-64	66	399	0.142	0.016
			≥65	76	352	0.178	0.018
		Central/South Asian	<45	99	663	0.130	0.012
			45-49	115	654	0.150	0.013
			50-54	177	634	0.218	0.015
			55-59	142	599	0.192	0.014
			60-64	145	513	0.220	0.016
			≥65	122	330	0.270	0.021
		East Asian	<45	34	318	0.097	0.016
			45-49	42	348	0.108	0.016
			50-54	44	320	0.121	0.017
			55-59	59	304	0.163	0.019
			60-64	41	185	0.181	0.026
			≥65	30	111	0.213	0.034
		European	<45	3182	18691	0.145	0.002
			45-49	5301	24346	0.179	0.002
			50-54	7596	28036	0.213	0.002
			55-59	10557	32474	0.245	0.002
			60-64	15638	42303	0.270	0.002
			≥65	13128	29616	0.307	0.002
		Middle Eastern	<45	<20	<200	0.118	0.026
			45-49	<20	<200	0.134	0.029
			50-54	27	97	0.218	0.037
			55-59	22	96	0.186	0.036
			60-64	21	74	0.221	0.043
			≥65	<20	<100	0.167	0.048
	Male	Ancestry-combined	<45	3690	17277	0.176	0.003
			45-49	5734	20079	0.222	0.003
			50-54	8110	21256	0.276	0.003
			55-59	12192	23746	0.339	0.002
			60-64	19765	30725	0.391	0.002
			≥65	19599	23772	0.452	0.002
			<45	<20	<100	0.131	0.037

© 2025. He J et al. Published online at http://www.aginganddisease.org/EN/10.14336/AD.2025.0754

		Admixed American	45-49	<20	<100	0.130	0.038
			50-54	<20	<100	0.250	0.065
			55-59	<20	<100	0.257	0.074
			60-64	25	46	0.352	0.057
			≥65	<20	<100	0.225	0.066
		African	<45	51	523	0.089	0.012
			45-49	64	582	0.099	0.012
			50-54	63	500	0.112	0.013
			55-59	51	312	0.140	0.018
			60-64	44	251	0.149	0.021
			≥65	68	280	0.195	0.021
		Central/South Asian	<45	111	843	0.116	0.010
			45-49	124	752	0.142	0.012
			50-54	135	680	0.166	0.013
			55-59	203	614	0.248	0.015
			60-64	193	514	0.273	0.017
			≥65	266	480	0.357	0.018
		East Asian	<45	<20	<300	0.078	0.018
			45-49	31	186	0.143	0.024
			50-54	<20	<200	0.106	0.024
			55-59	24	114	0.174	0.032
			60-64	39	85	0.315	0.042
			≥65	27	63	0.300	0.048
		European	<45	3476	15451	0.184	0.003
			45-49	5477	18308	0.230	0.003
			50-54	7856	19784	0.284	0.003
			55-59	11868	22567	0.345	0.003
			60-64	19436	29728	0.395	0.002
			≥65	19199	22862	0.456	0.002
		Middle Eastern	<45	24	187	0.114	0.022
			45-49	28	184	0.132	0.023
			50-54	28	115	0.196	0.033
			55-59	37	113	0.247	0.035
			60-64	28	101	0.217	0.036
			≥65	30	56	0.349	0.051
AoU	Sex-combined	Ancestry-combined	<45	5705	110491	0.049	0.001
			45-49	2186	24020	0.083	0.002
			50-54	3038	27300	0.100	0.002
			55-59	4369	31167	0.123	0.002
			60-64	6387	34305	0.157	0.002
			≥65	38522	91723	0.296	0.001
		African	<45	858	20510	0.040	0.001
			45-49	320	5339	0.057	0.003
			50-54	480	6662	0.067	0.003
			55-59	786	8352	0.086	0.003
			60-64	1157	9133	0.112	0.003
			≥65	3391	14270	0.192	0.003
			<45	1328	30605	0.042	0.001

		Admixed American	45-49	456	5956	0.071	0.003
			50-54	552	6041	0.084	0.003
			55-59	725	5940	0.109	0.004
			60-64	903	5352	0.144	0.004
			≥65	2897	9034	0.243	0.004
		East Asian	<45	114	4309	0.026	0.002
			45-49	35	608	0.054	0.009
			50-54	52	682	0.071	0.009
			55-59	59	613	0.088	0.011
			60-64	64	575	0.100	0.012
			≥65	344	1476	0.189	0.009
		European	<45	3301	51572	0.060	0.001
			45-49	1332	11623	0.103	0.003
			50-54	1930	13475	0.125	0.003
			55-59	2762	15880	0.148	0.003
			60-64	4206	18914	0.182	0.003
			≥65	31615	66191	0.323	0.001
		Middle Eastern	<45	32	655	0.047	0.008
			45-49	<20	<100	0.150	0.034
			50-54	<20	<100	0.033	0.019
			55-59	<20	<100	0.124	0.035
			60-64	<20	<100	0.162	0.036
			≥65	94	228	0.292	0.025
		Central/South Asian	<45	72	2840	0.025	0.003
			45-49	26	398	0.061	0.012
			50-54	21	352	0.056	0.012
			55-59	26	304	0.079	0.015
			60-64	40	243	0.141	0.021
			≥65	181	524	0.257	0.016
	Female	Ancestry-combined	<45	3845	74822	0.049	0.001
			45-49	1440	16106	0.082	0.002
			50-54	1939	17789	0.098	0.002
			55-59	2571	19571	0.116	0.002
			60-64	3629	20847	0.148	0.002
			≥65	17881	53754	0.250	0.002
		Admixed American	<45	866	21294	0.039	0.001
			45-49	308	4147	0.069	0.004
			50-54	346	4062	0.078	0.004
			55-59	453	3882	0.104	0.005
			60-64	552	3428	0.139	0.005
			≥65	1578	5756	0.215	0.005
		African	<45	585	13516	0.041	0.002
			45-49	225	3474	0.061	0.004
			50-54	335	4061	0.076	0.004
			55-59	478	4772	0.091	0.004
			60-64	680	5030	0.119	0.004
			≥65	2077	8424	0.198	0.004
			<45	40	1613	0.024	0.004

© 2025. He J et al. Published online at http://www.aginganddisease.org/EN/10.14336/AD.2025.0754

		Central/South Asian	45-49	<20	<300	0.068	0.016
			50-54	<20	<200	0.063	0.018
			55-59	<20	<200	0.086	0.021
			60-64	22	128	0.147	0.029
			≥65	63	245	0.205	0.023
		East Asian	<45	80	2814	0.028	0.003
			45-49	24	409	0.055	0.011
			50-54	35	419	0.077	0.013
			55-59	41	379	0.098	0.014
			60-64	37	373	0.090	0.014
			≥65	184	874	0.174	0.012
		European	<45	2259	35201	0.060	0.001
			45-49	859	7801	0.099	0.003
			50-54	1209	9014	0.118	0.003
			55-59	1580	10329	0.133	0.003
			60-64	2329	11843	0.164	0.003
			≥65	13949	38342	0.267	0.002
		Middle Eastern	<45	<20	<400	0.038	0.010
			45-49	<20	<100	0.123	0.041
			50-54	<20	<100	0.036	0.025
			55-59	<20	<100	0.075	0.036
			60-64	<20	<100	0.167	0.051
			≥65	30	113	0.210	0.034
	Male	Ancestry-combined	<45	1860	35669	0.050	0.001
			45-49	746	7914	0.086	0.003
			50-54	1099	9511	0.104	0.003
			55-59	1798	11596	0.134	0.003
			60-64	2758	13458	0.170	0.003
			≥65	20641	37969	0.352	0.002
		Admixed American	<45	462	9311	0.047	0.002
			45-49	148	1809	0.076	0.006
			50-54	206	1979	0.094	0.006
			55-59	272	2058	0.117	0.007
			60-64	351	1924	0.154	0.008
			≥65	1319	3278	0.287	0.007
		African	<45	273	6994	0.038	0.002
			45-49	95	1865	0.048	0.005
			50-54	145	2601	0.053	0.004
			55-59	308	3580	0.079	0.004
			60-64	477	4103	0.104	0.005
			≥65	1314	5846	0.184	0.005
		Central/South Asian	<45	32	1227	0.025	0.004
			45-49	<20	<200	0.053	0.016
			50-54	<20	<200	0.049	0.016
			55-59	<20	<200	0.071	0.021
			60-64	<20	<200	0.135	0.030
			≥65	118	279	0.297	0.023
		East Asian	<45	34	1495	0.022	0.004

© 2025. He J et al. Published online at http://www.aginganddisease.org/EN/10.14336/AD.2025.0754

			45-49	<20	<300	0.052	0.015
			50-54	<20	<300	0.061	0.014
			55-59	<20	<300	0.071	0.016
			60-64	27	202	0.118	0.021
			≥65	160	602	0.210	0.015
		European	<45	1042	16371	0.060	0.002
			45-49	473	3822	0.110	0.005
			50-54	721	4461	0.139	0.005
			55-59	1182	5551	0.176	0.005
			60-64	1877	7071	0.210	0.004
			≥65	17666	27849	0.388	0.002
		Middle Eastern	<45	<20	<300	0.059	0.014
			45-49	<20	<100	0.188	0.056
			50-54	<20	<100	0.029	0.028
			55-59	<20	<100	0.194	0.066
			60-64	<20	<100	0.157	0.051
			≥65	64	115	0.358	0.036

Supplementary Table 8. Chi-square trend test for age trends in the prevalence of hearing loss. UKB: UK Biobank; AoU: All of Us Research Program.

Variable tested	Cohort	Sex	Ancestry	Statistic	P
Age trend	UKB	Sex-combined	Ancestry-combined	11100.61	**<1E-300**
			African	53.09	**3.2E-13**
			East Asian	55.40	**9.8E-14**
			Admixed American	26.24	**3.0E-07**
			Central/South Asian	226.25	**3.9E-51**
			Middle Eastern	27.86	**1.3E-07**
			European	10018.83	**<1E-300**
		Female	Ancestry-combined	3479.42	**<1E-300**
			African	25.22	**5.1E-07**
			East Asian	20.22	**6.9E-06**
			Admixed American	16.24	**5.6E-05**
			Central/South Asian	43.51	**4.2E-11**
			Middle Eastern	4.05	0.044
			European	3158.01	**<1E-300**
		Male	Ancestry-combined	7680.30	**<1E-300**
			African	28.39	**9.9E-08**
			East Asian	39.67	**3.0E-10**
			Admixed American	10.10	**0.001**
			Central/South Asian	196.70	**1.1E-44**
			Middle Eastern	26.78	**2.3E-07**
			European	6884.56	**<1E-300**
	AoU	Sex-combined	Ancestry-combined	27919.51	**<1E-300**
			African	2466.14	**<1E-300**
			East Asian	476.59	**1.2E-105**
			Admixed American	3864.84	**<1E-300**
			Central/South Asian	425.19	**1.8E-94**

© 2025. He J et al. Published online at http://www.aginganddisease.org/EN/10.14336/AD.2025.0754

			Middle Eastern	110.70	**6.9E-26**
			European	16095.20	**<1E-300**
		Female	Ancestry-combined	12894.88	**<1E-300**
			African	1594.15	**<1E-300**
			East Asian	246.62	**1.4E-55**
			Admixed American	2176.15	**<1E-300**
			Central/South Asian	160.21	**1.0E-36**
			Middle Eastern	38.32	**6.0E-10**
			European	6960.68	**<1E-300**
		Male	Ancestry-combined	13751.79	**<1E-300**
			African	899.27	**1.4E-197**
			East Asian	227.78	**1.8E-51**
			Admixed American	1589.34	**<1E-300**
			Central/South Asian	252.44	**7.6E-57**
			Middle Eastern	63.26	**1.8E-15**
			European	8069.44	**<1E-300**

© 2025. He J et al. Published online at http://www.aginganddisease.org/EN/10.14336/AD.2025.0754

SUPPLEMENTARY DATA

Supplementary Table 9. Age-specific prevalence of hearing loss in UK Biobank (UKB) and All of Us Research Program (AoU) for additional age groups. The UKB participants were limited to 37-73 years.

Age, years	Female				Male			
UKB	EUR (n = 230868)	Non-EUR (n = 11380)			EUR (n = 196012)	Non-EUR (n = 9933)		
<45	0.145 (0.141-0.150)	0.104 (0.091-0.117)			0.184 (0.178-0.189)	0.105 (0.092-0.118)		
45-49	0.179 (0.174-0.183)	0.128 (0.114-0.141)			0.230 (0.225-0.236)	0.127 (0.112-0.141)		
50-54	0.213 (0.209-0.217)	0.162 (0.147-0.177)			0.284 (0.279-0.290)	0.147 (0.130-0.164)		
55-59	0.245 (0.241-0.249)	0.167 (0.150-0.184)			0.345 (0.340-0.350)	0.216 (0.195-0.236)		
60-64	0.270 (0.266-0.274)	0.193 (0.173-0.212)			0.395 (0.391-0.400)	0.248 (0.225-0.271)		
65-69	0.306 (0.301-0.310)	0.225 (0.200-0.249)			0.456 (0.451-0.461)	0.305 (0.280-0.330)		
≥70	0.358 (0.329-0.387)	0.242 (0.096-0.389)			0.469 (0.440-0.499)	0.314 (0.186-0.441)		
AoU	EUR (n = 134715)	AFR (n = 43657)	AMR (n = 46672)	Other (n = 9150)	EUR (n = 88086)	AFR (n = 27601)	AMR (n = 23117)	Other (n = 6215)
<45	0.060 (0.058-0.063)	0.041 (0.038-0.045)	0.039 (0.037-0.042)	0.027 (0.023-0.032)	0.060 (0.056-0.063)	0.038 (0.033-0.042)	0.047 (0.043-0.051)	0.027 (0.021-0.033)
45-49	0.099 (0.093-0.105)	0.061 (0.053-0.069)	0.069 (0.062-0.077)	0.066 (0.048-0.084)	0.110 (0.101-0.119)	0.048 (0.039-0.058)	0.076 (0.064-0.087)	0.067 (0.044-0.090)
50-54	0.118 (0.112-0.125)	0.076 (0.068-0.084)	0.078 (0.071-0.086)	0.070 (0.051-0.089)	0.139 (0.130-0.149)	0.053 (0.044-0.061)	0.094 (0.082-0.107)	0.054 (0.034-0.074)
55-59	0.133 (0.127-0.139)	0.091 (0.083-0.099)	0.104 (0.095-0.114)	0.093 (0.070-0.115)	0.176 (0.166-0.185)	0.079 (0.071-0.088)	0.117 (0.104-0.130)	0.081 (0.056-0.107)
60-64	0.164 (0.158-0.170)	0.119 (0.111-0.127)	0.139 (0.128-0.149)	0.111 (0.086-0.136)	0.210 (0.201-0.218)	0.104 (0.095-0.113)	0.154 (0.139-0.169)	0.128 (0.096-0.161)
65-69	0.196 (0.190-0.203)	0.151 (0.141-0.161)	0.165 (0.152-0.178)	0.141 (0.112-0.170)	0.268 (0.260-0.276)	0.132 (0.121-0.144)	0.207 (0.189-0.225)	0.160 (0.125-0.195)
70-74	0.244 (0.237-0.250)	0.188 (0.174-0.202)	0.213 (0.196-0.231)	0.161 (0.127-0.194)	0.336 (0.327-0.344)	0.186 (0.169-0.202)	0.275 (0.251-0.299)	0.232 (0.190-0.275)
75-79	0.299 (0.291-0.308)	0.255 (0.233-0.277)	0.269 (0.244-0.295)	0.229 (0.179-0.279)	0.442 (0.433-0.451)	0.269 (0.239-0.298)	0.402 (0.368-0.435)	0.313 (0.259-0.366)
80-84	0.348 (0.336-0.360)	0.338 (0.302-0.373)	0.280 (0.241-0.319)	0.255 (0.182-0.329)	0.492 (0.479-0.504)	0.323 (0.276-0.370)	0.402 (0.350-0.455)	0.342 (0.267-0.417)
≥85	0.463 (0.445-0.480)	0.373 (0.326-0.421)	0.460 (0.402-0.518)	0.333 (0.229-0.438)	0.575 (0.559-0.590)	0.424 (0.354-0.494)	0.452 (0.382-0.521)	0.482 (0.376-0.589)

SUPPLEMENTARY DATA

Supplementary Table 10. Sample overlap between different UK Biobank data sources and sex- and ancestry-specific hearing loss prevalence defined by first occurrence data in UK Biobank.

**Data source**		**First occurrences**	
		**Control**	**Case**
**Self-reported**	**Control**	305817	6516
	**Case**	100784	18183

**Data source**		**Hospital inpatient records**	
		**Control**	**Case**
**Self-reported**	**Control**	311562	834
	**Case**	114376	4604

**Sex**	**Ancestry**	**Total**	**First occurrence cases**	**First occurrence controls**	**Crude prevalence rate**
Female	Admixed American	590	21	569	0.036
	African	3736	81	3655	0.022
	Central/South Asian	3845	164	3681	0.043
	East Asian	1684	40	1644	0.024
	European	221536	11138	210398	0.050
	Middle Eastern	619	29	590	0.047
Male	Admixed American	330	15	315	0.045
	African	2625	63	2562	0.024
	Central/South Asian	4567	244	4323	0.053
	East Asian	884	26	858	0.029
	European	190006	12839	177167	0.068
	Middle Eastern	878	39	839	0.044

© 2025. He J et al. Published online at http://www.aginganddisease.org/EN/10.14336/AD.2025.0754
